# A low-cost and portable wrist exoskeleton using EEG-sEMG combined strategy for prolonged active rehabilitation

**DOI:** 10.3389/fnbot.2023.1161187

**Published:** 2023-05-24

**Authors:** Shiqi Yang, Min Li, Jiale Wang, Zhilei Shi, Bo He, Jun Xie, Guanghua Xu

**Affiliations:** Department of Mechanical Engineering, Xi'an Jiaotong University, Xi'an, China

**Keywords:** brain-machine interfaces, machine learning for robot control, rehabilitation robotics, sEMG, muscle fatigue detection

## Abstract

**Introduction:**

Hemiparesis is a common consequence of stroke that severely impacts the life quality of the patients. Active training is a key factor in achieving optimal neural recovery, but current systems for wrist rehabilitation present challenges in terms of portability, cost, and the potential for muscle fatigue during prolonged use.

**Methods:**

To address these challenges, this paper proposes a low-cost, portable wrist rehabilitation system with a control strategy that combines surface electromyogram (sEMG) and electroencephalogram (EEG) signals to encourage patients to engage in consecutive, spontaneous rehabilitation sessions. In addition, a detection method for muscle fatigue based on the Boruta algorithm and a post-processing layer are proposed, allowing for the switch between sEMG and EEG modes when muscle fatigue occurs.

**Results:**

This method significantly improves accuracy of fatigue detection from 4.90 to 10.49% for four distinct wrist motions, while the Boruta algorithm selects the most essential features and stabilizes the effects of post-processing. The paper also presents an alternative control mode that employs EEG signals to maintain active control, achieving an accuracy of approximately 80% in detecting motion intention.

**Discussion:**

For the occurrence of muscle fatigue during long term rehabilitation training, the proposed system presents a promising approach to addressing the limitations of existing wrist rehabilitation systems.

## 1. Introduction

Hemiparesis is a common sequala of stroke survivors (Wist et al., 2016), resulting in paralysis of one side of the body, including motor dysfunction and muscle weakness in the extremity (Maria and Eng, 2003; Li et al., 2013). Active therapy initiated by voluntary intention of patient is more effective for neuron recovery than continuous passive motion (CPM) (Takahashi et al., 2008), meanwhile, the repeated, continuous training can enhance the functional recovery of stroke patients (Kwakkel et al., 2004; Wang et al., 2013). Since wrist joint is critical for activities of daily living (ADLs), many rehabilitation systems were proposed to assist the active training of wrist (Krebs et al., 2007; Song et al., 2013; Abdallah et al., 2016; Lin et al., 2020). However, in terms of hemiparesis rehabilitation, these systems possess two insufficiencies. First, these rehabilitation robots are controlled by the paretic arm, which is not applicable for the acute hemiparesis patients because of the motor dysfunction of their damaged side. Second, the above methods neglect the occurrence of muscle fatigue during the training, causing a risk of impact on the active participance of the patients.

Bilateral control is naturally appropriate for hemiparesis rehabilitation since it utilizes surface electromyography (sEMG) signals of the healthy arm to manipulate the movement of the affected one, and it has been employed in upper limb rehabilitation controlling (Nielsen et al., 2011; Leonardis et al., 2015). However, sEMG-based control encounters challenges of muscle fatigue due to the increasing workload of the unimpaired side caused by the habit of the patients with hemiparesis, in which they tend to rely on their healthy extremity instead of the impaired one during ADLs (Wolf et al., 1989). When the muscle becomes fatigued, the physical characteristics of sEMG change (Viitasalo and Komi, 1997; Dimitrova and Dimitrov, 2003; Lalitharatne et al., 2013), making bilateral control inaccurate and unstable. In that case, the control issues and discomfort can reduce patients' willingness to engage in rehabilitation, leading to fragmented and segmented rehabilitation that violates the principles of prolonged and consecutive rehabilitation. Researchers have studied the detection of muscle fatigue during rehabilitation training Shahmoradi et al. (2017) extracted time and frequency domain features and used Hidden Markov Model to fatigue status recognition system in post-stroke rehabilitation exercises; Mugnosso et al. (2018) designed an indicator based on mean frequency of sEMG to detect the ongoing fatigue during repeated training; Moreover, other studies also employed machine learning methods based on time and frequency features to evaluate muscle fatigue, but they have not proposed an applicable solution for the training after muscle fatigue (Gerdle et al., 2000; De et al., 2018).

EEG is commonly used to control robots or devices in many studies since it provides a direct representation of brain activity (Edelman et al., 2019; Li et al., 2019). However, its accuracy is less than that of normal sEMG signals, which are recorded when there is no muscle fatigue (Li et al., 2017). Consequently, researchers (Lalitharatne et al., 2013; Chowdhury et al., 2019; Edelman et al., 2019; Li et al., 2019) have attempted to enhance control performance by combining EEG and sEMG signals. Nevertheless, the requirement for sophisticated and costly devices with numerous electrodes and wires, which necessitate the application of conductive gels, presents a challenge for combining control. Conversely, ideal human-machine interfaces (HMIs) should be low-cost, lightweight, and user-friendly (Mahmood et al., 2021).

To overcome these challenges, this paper proposes a low-cost, portable, and user-friendly system for active wrist rehabilitation. The system's core innovation is a control strategy that switches the control mode from sEMG to EEG when muscle fatigue occurs. The aim is to maintain coherent and consistent bilateral training while minimizing accuracy loss and discomfort. The main contribution of this paper is a muscle fatigue detection model that has a post-processing layer embedded with CNN. The Boruta algorithm is employed to select the optimal features. The Boruta algorithm is a feature selection approach based on Random Forest classification (Kursa and Rudnicki, 2010; Ahmadizadeh et al., 2019), which provides unbiased and stable selection of constructive features. The paper also proposes an alternative control mode that uses EEG signals to maintain active bilateral control. Finally, evaluation experiments are conducted to validate the optimal setting of the fatigue detection method and the performance of the EEG control mode.

The present paper is structured as follows: Section Methodology provides a detailed account of the employed methods in the proposed system. Section Experiments and protocols outlines the experimental protocol, while the results obtained are presented and analyzed in Section Experiment results. The ensuing discussion and interpretation of the study's findings are included in Section Discussion. Finally, Section Conclusion summarizes the main conclusions drawn from this investigation.

## 2. Methodology

### 2.1. Overall design of EEG and sEMG control system

**Figure 1:**
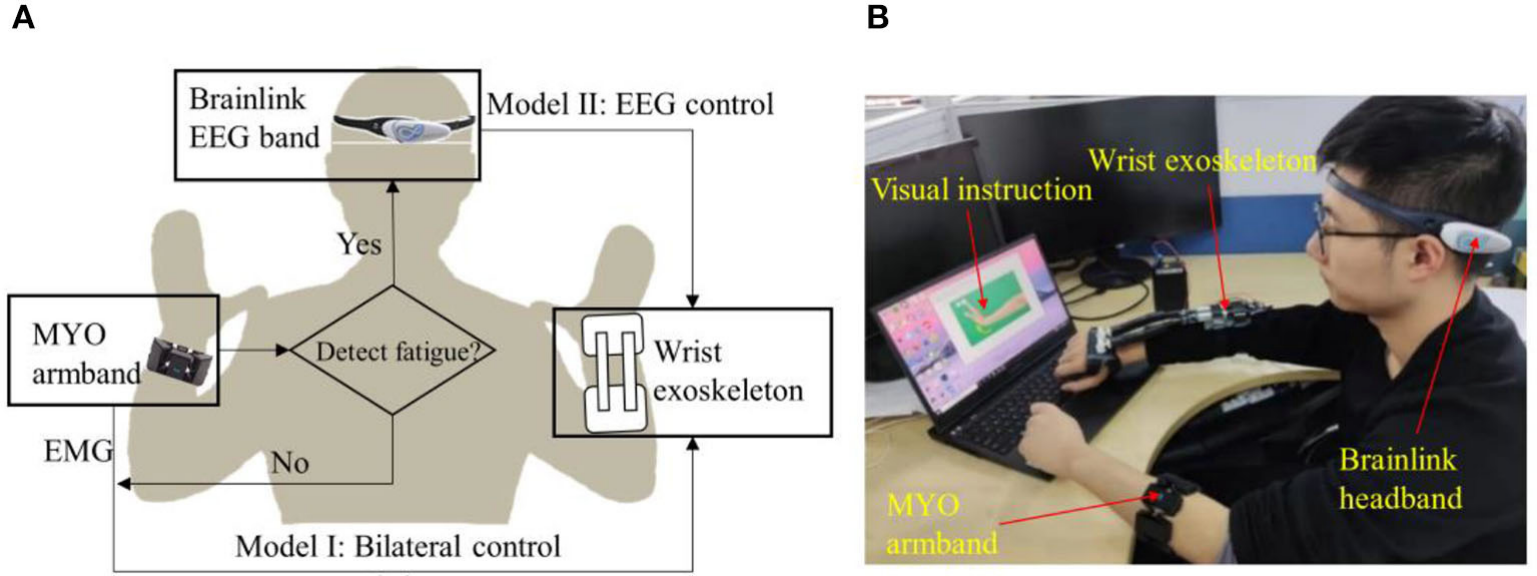
Overview of the system. **(A)** Sketch of exoskeleton-assisted wrist rehabilitation using a hybrid control strategy with low-cost EEG and sEMG sensors. **(B)** Integration and application of the system.

**Figure 2:**
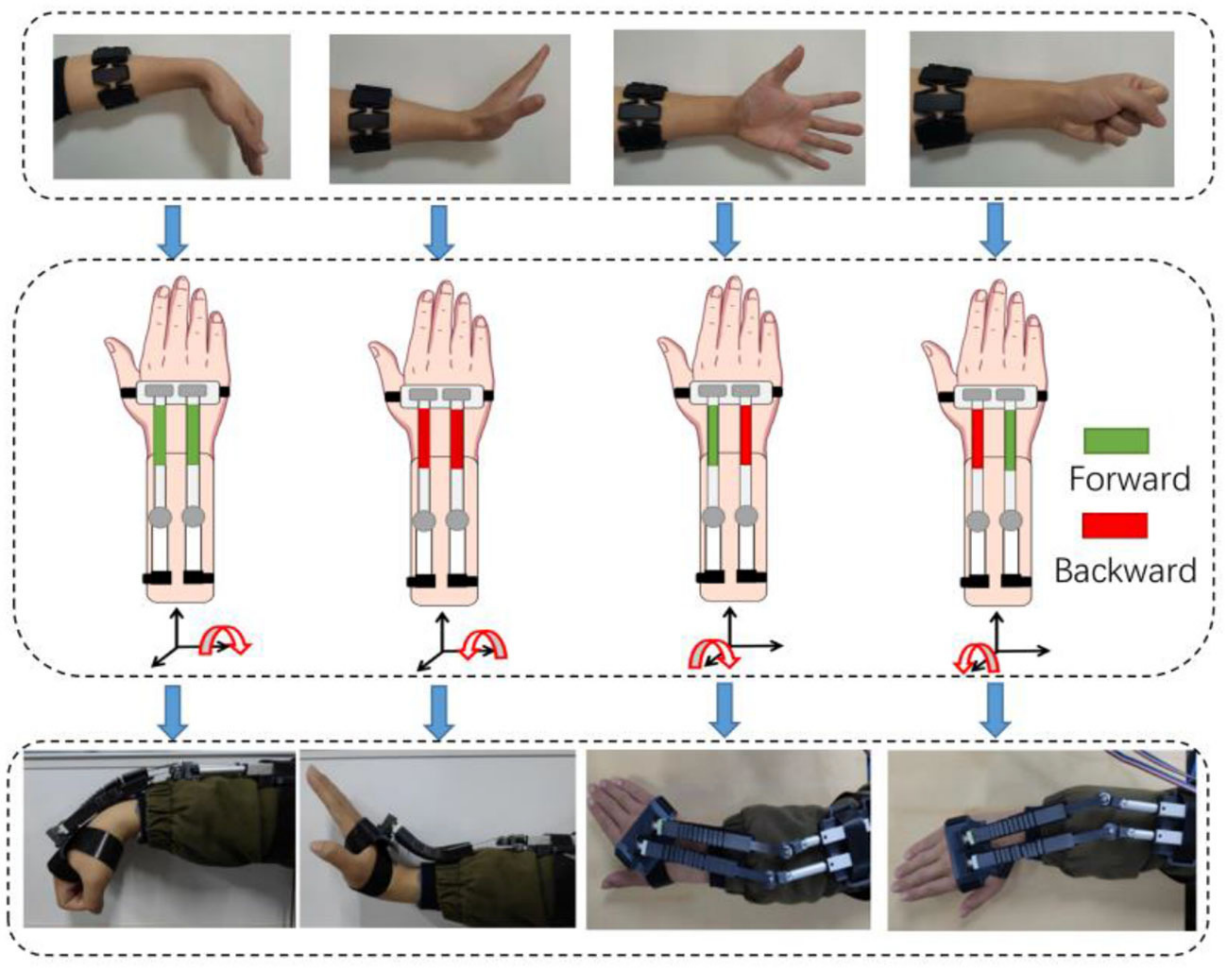
Diagram of bilateral control for four motions by sEMG. The interconnections are: wrist flexion-wrist flexion, wrist extension-wrist extension, fist clenching-radial deviation and five fingers opening-ulnar deviation. The green block corresponds to the forward motion of the linear motor, while the red block denotes the backward motion. A combination of different distances can generate four types of movements.

In this paper, a system comprising two modes is presented, namely sEMG Mode (Mode I) and EEG Mode (Mode II), as illustrated in Figure 1A. Mode I utilizes surface electromyography (sEMG) data acquired through a wireless MYO Armband (Thalmic Labs., Canada) to detect and interpret the user's intended motion, and Mode II works as an alternative of Mode I when muscle fatigue occurs. The MYO Armband is capable of recognizing six types of gestures, which are transmitted to the computer as integer values via Bluetooth at a frequency of 50 Hz. Then the computer activates the exoskeleton, which assists in executing the intended motion, as depicted in Figure 2. It should be noted that the system does not initiate detection of the next gesture until the ongoing motion has been completed. Moreover, a fatigue detection program runs concurrently with the motion control program in Mode I, enabling real-time monitoring of muscle fatigue. Once fatigue is detected, gesture recognition is halted and the system switches to Mode II.

Mode II utilizes a low-cost and wireless headband, Brainlink (Macrotellect Ltd., Shenzhen, China), to capture EEG signals. Brainlink is a lightweight (39 g) and affordable (< 120 USD) consumer EEG headband that utilizes dry electrodes, thereby eliminating the need for conductive gels, reducing noise (Mahmood et al., 2021), and shortening preparation time. The system detects the user's state of movement or non-movement via EEG and activates the exoskeleton to perform the rehabilitation movements accordingly when movement is detected, as depicted in Figure 1B.

The wrist exoskeleton is a soft-rigid combined assistance device designed in a prior study (Yang et al., 2021) and characterized by portability, weighing only 268 g without circuits and battery. Another advantage is the adaptable soft actuator, which minimizes the risk of secondary injury.

### 2.2. Acquisition, preprocessing and feature extraction of sEMG data

The sEMG signals are acquired through the eight electrodes around MYO Armband at 200 Hz. The armband is placed centrally between the extensor carpi radialis and extensor digitorum muscles and is fitted snugly to the user's arm. In the fatigue detection process, the sEMG signals are transmitted to a computer and are subsequently filtered using a fourth-order Butterworth bandpass filter with cutoff frequencies of 10 and 80 Hz, in addition to a notch filter at 50 Hz. Let *T* represents the acquisition time and *R*_*M*_ is the acquisition rate of sEMG, and the sEMG data *D*_*M*_ is obtained with a size of 8 × *T*·*R*_*M*_.

Next, a sliding window approach is applied to segment the EEG data into smaller slices for local feature extraction. The window length and step length are denoted by *T*_*w*_ and *T*_*s*_, respectively. For the sEMG data, a non-overlapping window with a length of *T*_*w*_ = *T*_*s*_ = 0.25*s* (roughly 50 samples) is used for segmentation. Within each window, features are extracted from the eight sEMG channels using four different schemes presented in this paper. Scheme A involves 13 features, including minimum, maximum, mean, and standard deviation of sEMG amplitude and frequency, as well as spectral entropy, spectral flux, zero crossing rate, entropy energy, and Wilson amplitude (WAMP). Scheme B is similar to scheme A, except for the replacement of mean frequency with median frequency (MDF). Scheme C includes eight features, namely root mean square (RMS), MDF, skewness, kurtosis, form factor, crest factor, impulse factor, and margin factor. Finally, scheme D comprises only two features, RMS and MDF. As each channel's features are computed within one window, a basic feature matrix *M*_*B*_ of size *F*_*M*_×8. is obtained. To construct the final feature matrix *M*_*M*_, three adjacent matrices *M*_*B*_ are concatenated, resulting in a size of 3*F*_*M*_×8. The dataset of *M*_*M*_ is denoted by *DS*_*M*_, which has a size of *N*_*M*_×3*F*_*M*_×10, where *N*_*M*_ = [(*T*·*R*_*M*_−*T*_*w*_)/*T*_*s*_+1 ]/3.

### 2.3. Acquisition, preprocessing and feature extraction of EEG data

Brainlink utilizes three dry electrodes located on the forehead to generate a bipolar EEG channel corresponding to F7–Fp1 (Japaridze et al., 2022). The sampling rate of the Brainlink headband is 512 Hz and it employs the NeuroSky ThinkGear ASIC Module (NeuroSky, USA) to calculate the power spectral density (PSD) values of the raw EEG data every second. The PSD values are sorted into eight distinct frequency bands, including Delta, Theta, Low alpha, High alpha, Low beta, High beta, Low gamma, and Middle gamma. The software development kit used for this purpose is MATLAB (MathWorks, USA), which reads these values as hexadecimal numbers. These hexadecimal PSD values are then normalized to a range of [0, 1] by dividing the PSD value of each band by the total PSD values of the corresponding row. Next, the hexadecimal PSD value of each band is converted into decimal number, and the PSD value of each band is divided by the total PSD values of the row to normalize the data into [0, 1]. Let *R*_*E*_ denote the acquisition rate of EEG. The EEG data *D*_*E*_ has a size of 8 × *T*·*R*_*E*_.

To crop the EEG data *D*_*E*_, *T*_*w*_ and *T*_*s*_ are set as 8 and 2 s, respectively, according to the experiments reported in section EEG classification results. The sliding window moves along the temporal axis, and the EEG feature matrix *M*_*E*_ is computed within each window. Here, 10 features are considered for EEG classification, which are the eight normalized PSD values in *D*_*E*_ plus the values of β/α and β/(α+θ). Because β/α and β/(α+θ) can show the attention level of a subject (Zhang, 2018). These two features are computed using the normalized PSD values in *D*_*E*_, and the EEG feature matrix *M*_*E*_ has a size of *T*_*w*_×10. Let *DS*_*E*_ represents the dataset of *M*_*E*_, *DS*_*E*_ has a size _*N*_*E*_×*Tw*_×10, where *N*_*E*_ = (*T*·*R*_*E*_−*T*_*w*_)/*T*_*s*_+ 1.

### 2.4. Muscle fatigue detection using CNN with boruta and post-processing

**Figure 3:**
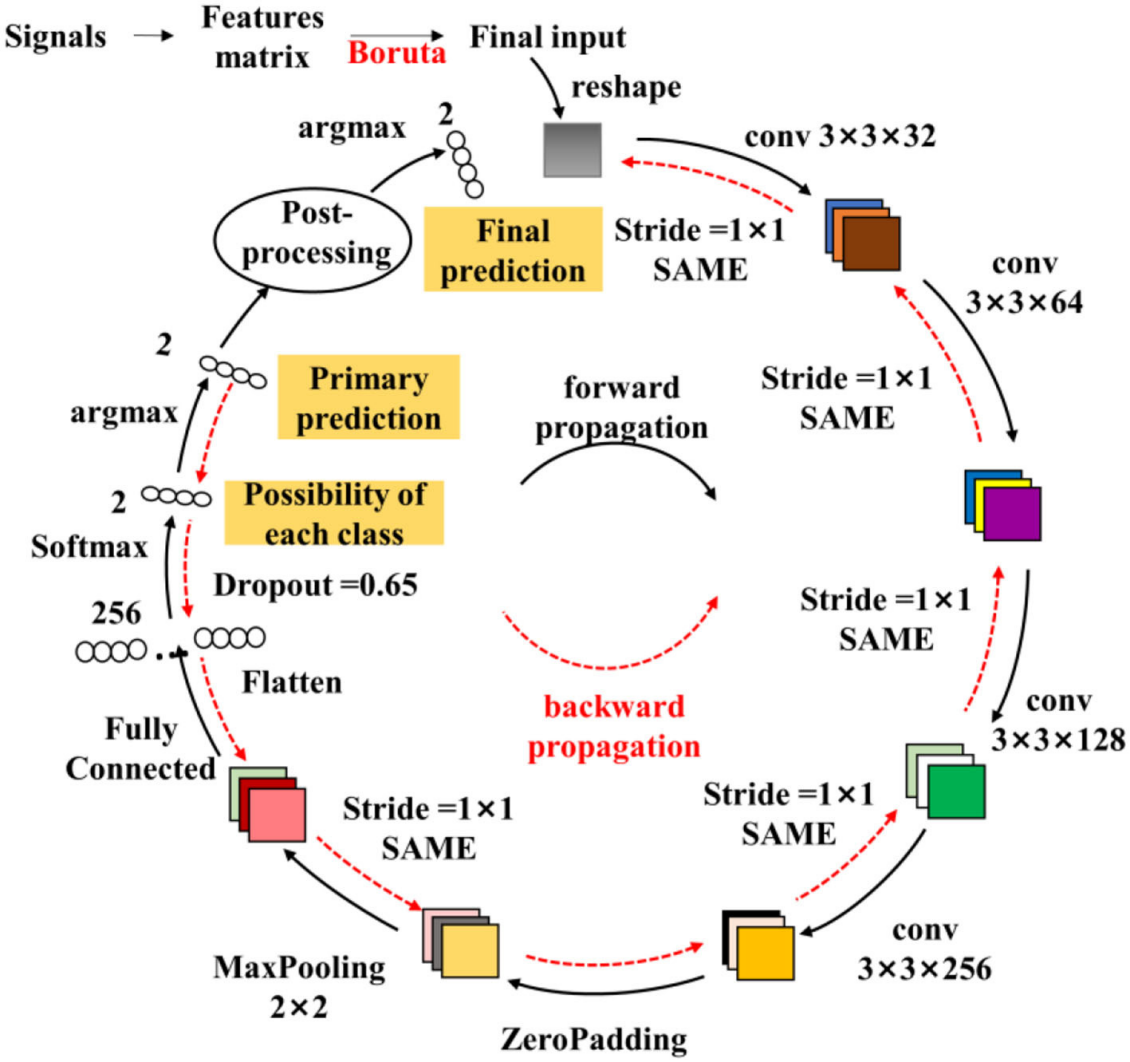
Structure of the fatigue-CNN used in fatigue detection. The colored squares represent the output of the convolutional layers, and the numbers nearby are the size of the corresponding filters. The strides and padding method are listed as well. And the small ellipses are the 1-D features.

To monitor the muscle fatigue during rehabilitation, a model named fatigue-CNN with Boruta-based feature selection has been developed. As illustrated in Figure 3, this model consists of a post-processing layer and four convolutional layers. The fatigue-CNN is trained at a learning rate of 0.0001 for 120 epochs, and batch size of 32. The layers are initialized using the Xavier method, and the fully-connected layers use a dropout rate of 0.65. The optimizer used is Adam, and the loss function is set to cross entropy. The fatigue detection process is as follows: First, the feature matrices *M*_*M*_ are normalized using min-max normalization, and then the most valuable features are selected using the Boruta algorithm. Next, the selected feature matrices are passed through the four convolutional layers to extract higher-level features and undergo Maxpooling. The resulting features are flattened, then passed through a softmax layer and argmax to obtain the primary prediction results. Finally, the possibility values of each class and the primary prediction results are input into the post-processing layer to produce the ultimate fatigue prediction.

The post-processing step takes into account the sequential relationship of sEMG to overcome the deficiency of the Conventional CNN mechanism which treats feature matrices as independent samples, thus neglecting the interconnections between the samples. For example, a real sEMG sequence may be [0 0 0 0 0 0 …1 1 1 1 1 1], where “1” represents fatigue and “0” denotes non-fatigue, but the neural network may output a result of [0 0 0 1 1 0…1 0 0 1 0 1]. To address this issue, a non-linear bias using neighboring information (cf. equation 1) is proposed to rectify the final recognition results. The detailed workflow is shown in 4.2.


(1) $$\begin{array}{l} f_{ci}=argmax\left(P_{i}^{m}+\frac{|R_{m}^{K}-P_{i}^{m}|\cdot \left(R_{m}^{K}-P_{i}^{m}\right)}{R_{m}^{K}}\right) \end{array}$$

where **f**_**ci**_ is the final classification result of *ith* input, $P_{i}^{m}$ is the classification possibility of the CNN softmax layer for the *ith* data and *m* class, *m*∈(0, 1) is the class of fatigue or non-fatigue And $R_{m}^{K}$ is the ratio of *m* class data in [*i-K*:*i*+*K*] adjacent range.

### 2.5. Active control based on EEG signal

To facilitate EEG-based control of the exoskeleton, an EEG-CNN model is proposed for recognizing the user's intended movements from EEG signals. The architecture of EEG-CNN is similar to that of the fatigue-CNN, with the exception of the absence of the post-processing layer. The EEG-CNN is trained using a learning rate of 0.0001 for 120 epochs with a batch size of 16. All layers are initialized with the Xavier initialization method, and the fully connected layers have a dropout of 0.65. The loss function is set to cross entropy, and the Adam optimizer is used for optimization.

As described in section Acquisition, preprocessing and feature extraction of EEG data, the feature matrices *M*_*E*_ have been normalized prior to input into the EEG-CNN. These matrices then pass through four convolutional layers, a Maxpooling layer, and fully connected layers to produce the final prediction of motion intention. The EEG-based classification consists of two categories: “has motion intention” or “none motion intention.” During EEG mode, the user wears the EEG headband, and the screen displays a series of wrist motions. After each prompt, if the user intends to perform the displayed motion, the user simply repeats the raising and lowering of the arm, after which the exoskeleton will perform the corresponding actions. Conversely, if the user does not wish to move, the user should keep their gaze fixed on the black cross and remain still.

## 3. Experiments and protocols

### 3.1. Experiments on sEMG control before and after muscle fatigue

**Figure 4:**
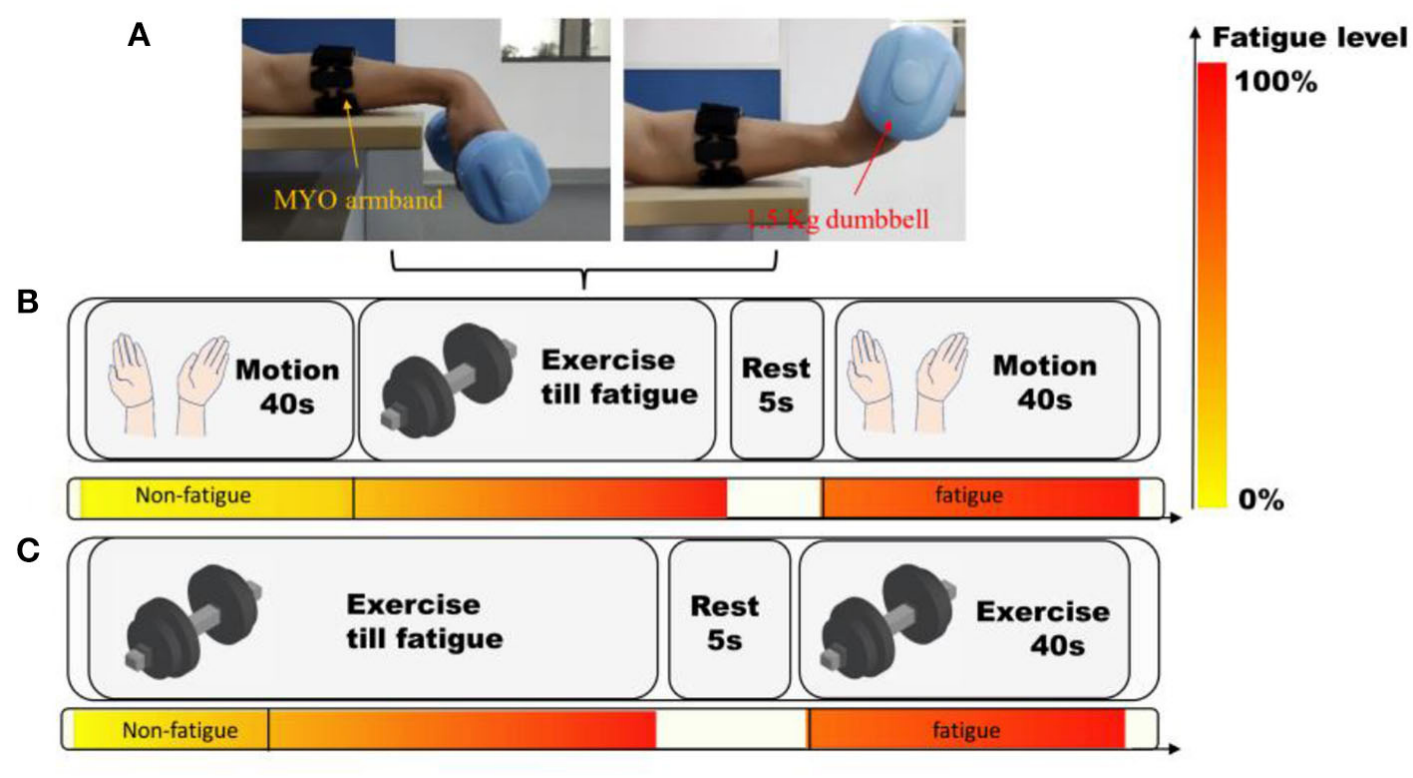
The protocol of the experiments using MYO armband. **(A)** The setting of MYO armband and subjects. **(B)** Experiments on hand motion recognition. **(C)** Acquisition of muscle fatigue data during wrist flexion and extension. The color-bar manifests the progress of muscle fatigue.

Eight subjects (two females and six males) with average age at 23.6 were involved in this experiment, and the protocol is shown in Figure 4B. The subjects seated straight and equipped with MYO armband and they followed the instructions on the screen to perform four hand motions using their left hands. For each subject, every motion was repeated for 20 times. Thus, 20 times × 8 subjects = 160 attempts and the corresponding exoskeleton motions were recorded. In total, four hand gestures were tested, which resulted in 640 trials in total.

Then, the subjects held a dumbbell of 1.5 kg, and kept doing wrist flexion and extension until they felt exhausted. Subsequently, they continuously grasped a grip until their fingers got tired as well. After the fatigue of both wrist and fingers, they rested for 20 s. Then they performed the four hand motions again, and another 640 hand motion recognition results were recorded. After the experiments, the subjective feelings of the subjects were inquired and recorded.

Eight subjects (two females and six males) with an average age of 23.6 participated in this experiment, which followed the protocol outlined in Figure 4B. The subjects seated upright and wore MYO armbands. Then they followed the instructions on the screen to perform four hand motions using their left hands. And each motion was repeated 20 times by every subject, resulting in a total of 160 attempts per subject, and 640 attempts in total across all subjects.

The experiment then proceeded to test the recognition of gesture after the fatigue of subjects. At first, they held a 1.5 kg dumbbell and continuously performed wrist flexion and extension until they felt exhausted. Then they grasped a grip until their fingers became fatigued. And the subjects rested for 20 s before performing the four hand motions again. A further 640 hand motion recognition results were recorded. Finally, the subjective feelings of the subjects were recorded and analyzed after the completion of the experiments.

### 3.2. Acquisition and construction of fatigue sEMG dataset

In order to evaluate the performance of the fatigue detection method, two datasets are involved, namely, a public sEMG dataset and a MYO dataset collected in this paper. The public dataset (Papakostas et al., 2019) contains sEMG data of three motions: shoulder flexion (SF), shoulder abduction (SA) and elbow extension (EE). All the signals were extracted in one channel with a sample rate of 1,926 Hz. Following the processing approach in 2.2, the validation dataset *DS*_*M*_ was obtained.

In this paper, a dataset of sEMG signals during constantly fulfilling wrist flexion and extension (WFE) were recorded. Four males and two females (not the same individuals in 3.1), who were all healthy students with an average age at 24.2, were recruited to collect the data. They seated on a chair in a quiet room, and putted the left arm on a horizontal desk wiring MYO Armband, as depicted in Figure 4A. The experimental procedure consisted of two trials, as shown in Figure 4C. In the first trial, participants were asked to perform WFE movements with a 1.5 kg dumbbell at a uniform speed until they felt significantly fatigued. After a 5 s break, they tried their best to continue repeating the movements for 40 s. The samples collected during the first 20 s of the trial were considered as “non-fatigue,” while the last 40 s of data were labeled as “fatigue.” All the chosen samples were labeled accordingly, except for one male subject who did not feel significant fatigue.

For both datasets, following the aforementioned procedures, the corresponding *Ds*_*M*_ was produced. It is noteworthy that the *Ds*_*M*_ was split by motion. All the feature matrices *M*_*M*_ of the same motion of all the subjects were merged in one *Ds*_*M*_, in which the *M*_*M*_ of certain subject were ordered by chronological sequence, and then catenated by subject. Finally, the number of *M*_*M*_ in *Ds*_*M*_ of SF, SA, EE, WFE were 954, 1,015, 901 and 998. Afterwards, the validation experiments were performed and the results are shown in Table 1. When dividing the EMG dataset, a special partition method was used to produce five-fold validation dataset, that was: the *ith* sample of each five samples was selected to form the testing dataset of in the *ith* fold, and the remaining formed the training dataset.

**Table 1:** The accuracies of muscle fatigue detection for different methods.

**Motion**	**Original CNN accuracy**	**Fatigue-CNN accuracy**				
		**K** = **3(%)**	**K** = **4(%)**	**K** = **5(%)**	Δ **(%)**	***P*** **-value**
SF	73.20 ± 0.72	81.16 ± 0.67	**81.64 ± 1.78**	77.21 ± 1.54	4.43	/
SA	63.73 ± 0.87	65.28 ± 2.28	65.55 ± 1.43	**68.63 ± 2.07**	3.35	/
EE	84.14 ± 0.49	89.84 ± 0.76	92.34 ± 0.64	**93.21 ± 0.80**	3.37	/
WFE	77.94 ± 0.98	86.21 ± 1.02	87.54 ± 1.30	**88.43 ± 1.79**	2.22	/
SF + Boruta	74.00 ± 1.00	81.56 ± 1.08	81.80 ± 1.07	**83.39 ± 1.48**	1.83	2.20E-03
SA + Boruta	65.72 ± 0.64	70.00 ± 1.86	**71.74 ± 1.87**	70.28 ± 1.98	0.74	8.76E-03
EE + Boruta	85.04 ± 0.48	92.73 ± 0.79	92.86 ± 0.98	**93.26 ± 0.91**	0.53	9.35E-01
WFE + Boruta	78.44 ± 0.99	86.53 ± 1.44	**88.62 ± 1.98**	88.14 ± 2.01	2.09	8.27E-01

The bold values in “K = 3, K = 4, K = 5” columns indicate the highest accuracy in the three columns.

### 3.3. Experiments on muscle fatigue detection methods using boruta and post-processing

The experiments of fatigue detection algorithm are split into two parts. In experiment I, the algorithm is validated on four motions with features of scheme A. In experiment II, the fatigue detection algorithm is tested with or without Boruta optimization with features of scheme B, C and D.

In experiment I, the aforementioned datasets of SA, EE, SF and WFE were processed through the procedures in 2.2 and extracted features through scheme A, and each motion produced one validation dataset *DS*_*M*_ which is Group 1. Then a copy of these datasets was processed by Boruta algorithm which is named Group 2. Then the samples in two groups were disordered and divided into testing data and training data at a ratio of 2:8. Afterwards, they were tested by original CNN and the fatigue-CNN through ten times of five-folds cross validations, while three K values were tested in fatigue-CNN group (K = 3, K = 4 and K = 5).

In experiment II, only WFE dataset was employed and B, C and D feature extraction schemes are used to generate *DS*_*M*_ (Group 1). A copy of each of the three *DS*_*M*_ was processed by Boruta algorithm (Group 2). Following the same procedures of experiment I, the testing and training datasets were produced and the two groups were tested by fatigue-CNN through ten times of five-folds cross validations with three K values.

### 3.4. EEG acquisition and experiments of movement state

Six subjects, four males and two females with an average age of 23.4, participated in this experiment. All the subjects were in good health and did not have any neural diseases. They were seated upright in a quiet room, maintaining a distance of approximately 40 cm from the screen and wearing an EEG headband on their forehead.

**Figure 5:**
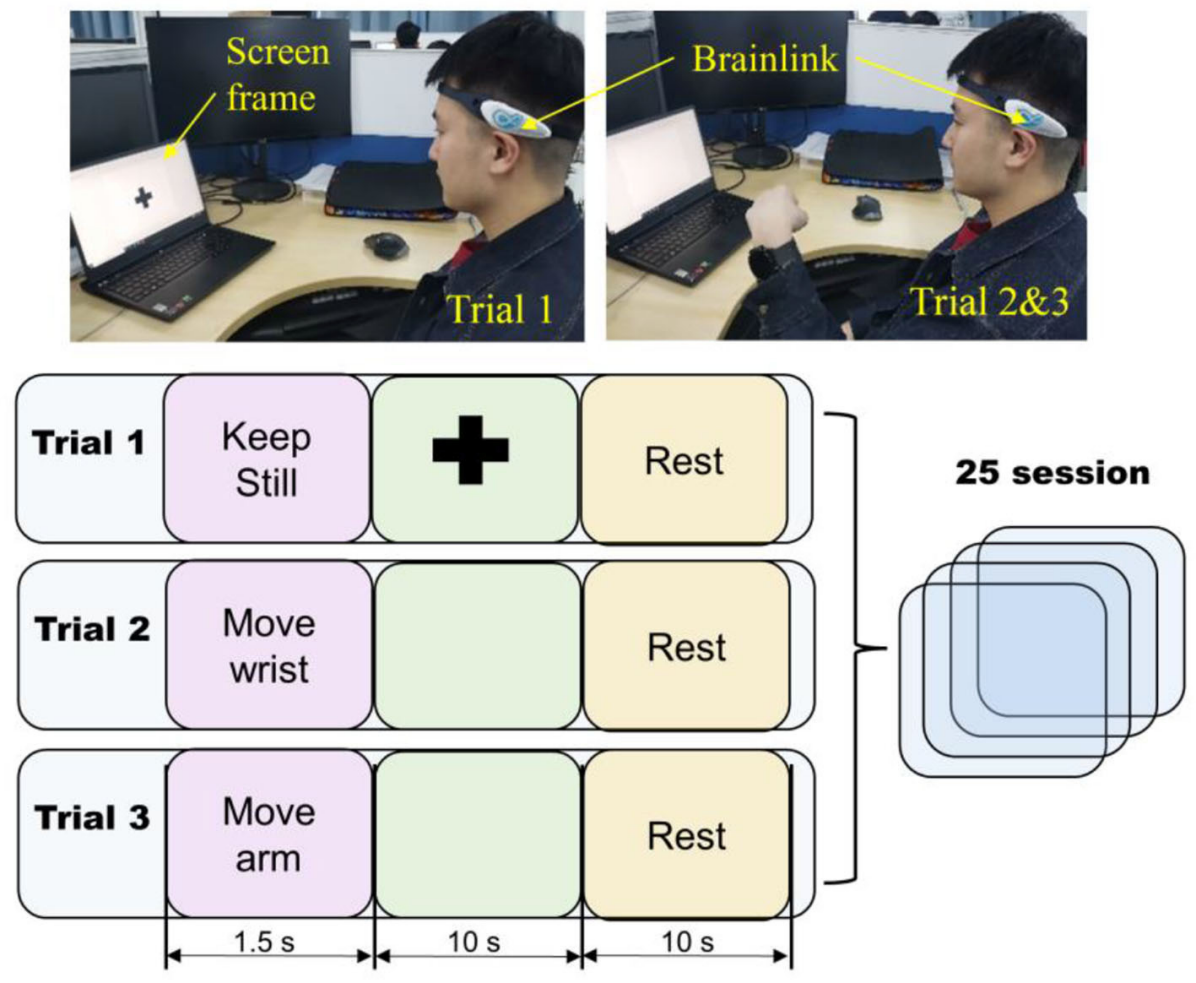
The EEG acquisition protocol. The light blue square represents one trial which contains three screen frames. The duration of each frame is shown under the corresponding squares.

For each subject the test protocol consisted of 30 sessions, and each session included three randomly selected trials, as illustrated in Figure 5. The first trial began with a text prompt instructing the subject to “keep still,” which was displayed for 1.5 s. Next, a black cross was displayed on the screen for 10 s, and the subject kept gazing on the cross and keeping their body still. In the second and third trials, the subject was instructed to perform a specific motion, either wrist flexion and extension or arm raising, after a 1.5 s prompt. There was no black cross on the screen during these trials, and the screen was completely black while the subject performed the motion. After each trial, the subject had a 10 s rest.

The samples of keeping still were labeled by “0.” meanwhile, the samples of wrist movements and arm movements were labeled by “1.” Various combinations of window and step length (*T*_*s*_ϵ[2, 4, 6, 8], *T*_*w*_ϵ[6, 8, 10] ) were tested to investigate the proper settings of EEG mode, and the corresponding *Ds*_*E*_ was produced. Notably, the *Ds*_*E*_ of all subjects were mixed, then ten times of five-folds cross validations were completed by EEG-CNN for the mixed dataset of all subjects.

## 4. Experiment results

### 4.1. Results of sEMG control before and after muscle fatigue

In the sEMG control experiments, a successful control was defined as: the exoskeleton was activated according to the bilateral control protocol in section Overall design of EEG and sEMG control system. The recognition results of the same motion of different subjects were merged, as a result, the successful control ratio for wrist flexion, wrist extension, fist clenching and five fingers opening are 97, 95, 95, and 89%, respectively.

In contrast, the accuracies of the four motions after fatigue are 95, 93, 82, and 80%, respectively. The accuracies of wrist flexion and wrist extension are almost the same, while those of fist clenching and fingers opening decrease by nearly 10%. In addition, 7 out of 8 subjects reported that they were not willing to continue doing movement and could not standardly fulfill the movement after fatigue.

### 4.2. Experimental results of fatigue detection method

The results of Experiment I are shown in Table 1, and it can be concluded into three aspects. First is the effect of the post-processing layer, which significantly enhances the accuracy of fatigue detection for all experimental groups. Specifically, the improved percentages are 4.90–10.49% for the datasets without Boruta, while the highest improvement is obtained in WFE group (10.49%), and the highest accuracy is observed in EE group (93.21 ± 0.80%). Meanwhile, in the Boruta groups, the EE + Boruta has the highest accuracy (93.26 ± 0.91%). However, the accuracy after post-processing is considerably influenced by the value of K. Basically, the accuracy increases with the rise of K, and the highest accuracies almost emerge in K = 5.

**Figure 6:**
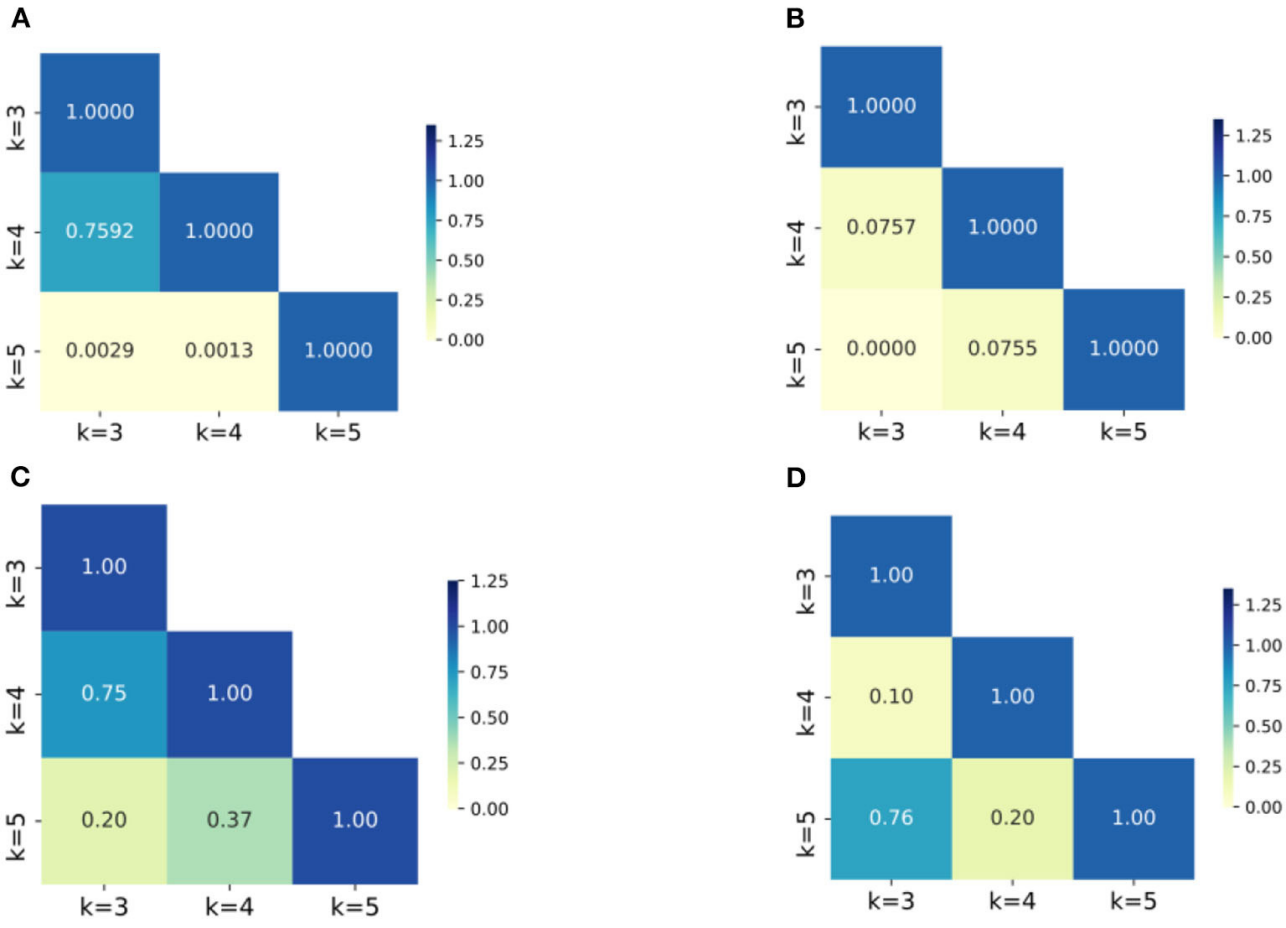
The heatmaps of P-values of the accuracies for groups with different K values. **(A)** SA dataset without Boruta; **(B)** EE dataset with Boruta; **(C)** SA dataset with Boruta; **(D)** EE dataset with Boruta; The numbers in the figures are the *P*-values of the corresponding groups and the color-bar visualizes the *P*-values.

Second, after applying the Boruta algorithm, the highest accuracies of SF and SA showed a significant improvement of 1.75 and 3.11%, respectively. Additionally, the validation results were tested and found to align with a normal distribution, and *T*-tests were conducted to determine the significance of the accuracies before and after applying the Boruta algorithm. The *P*-values for SF and SA were found to be 2.20E-03 and 8.76E-03, respectively, indicating a significant improvement in their accuracies. Furthermore, the Boruta algorithm was successful in reducing the fluctuations of accuracies for all four datasets. In SA and EE, for instance, the difference between the highest and lowest accuracies decreased to 0.74 and 0.53%, respectively, which are only one quarter of the original difference (3.35 and 3.37%). Heatmaps (a) and (b) in Figure 6 show the *P*-value of accuracies at different *K*-values for SA and EE, with the majority of *P*-values being close to or <0.05. Conversely, the *P*-values after applying Boruta were much higher than 0.05, indicating that the performances of accuracies were not significantly different. In other words, post-processing was more stable.

**Figure 7:**
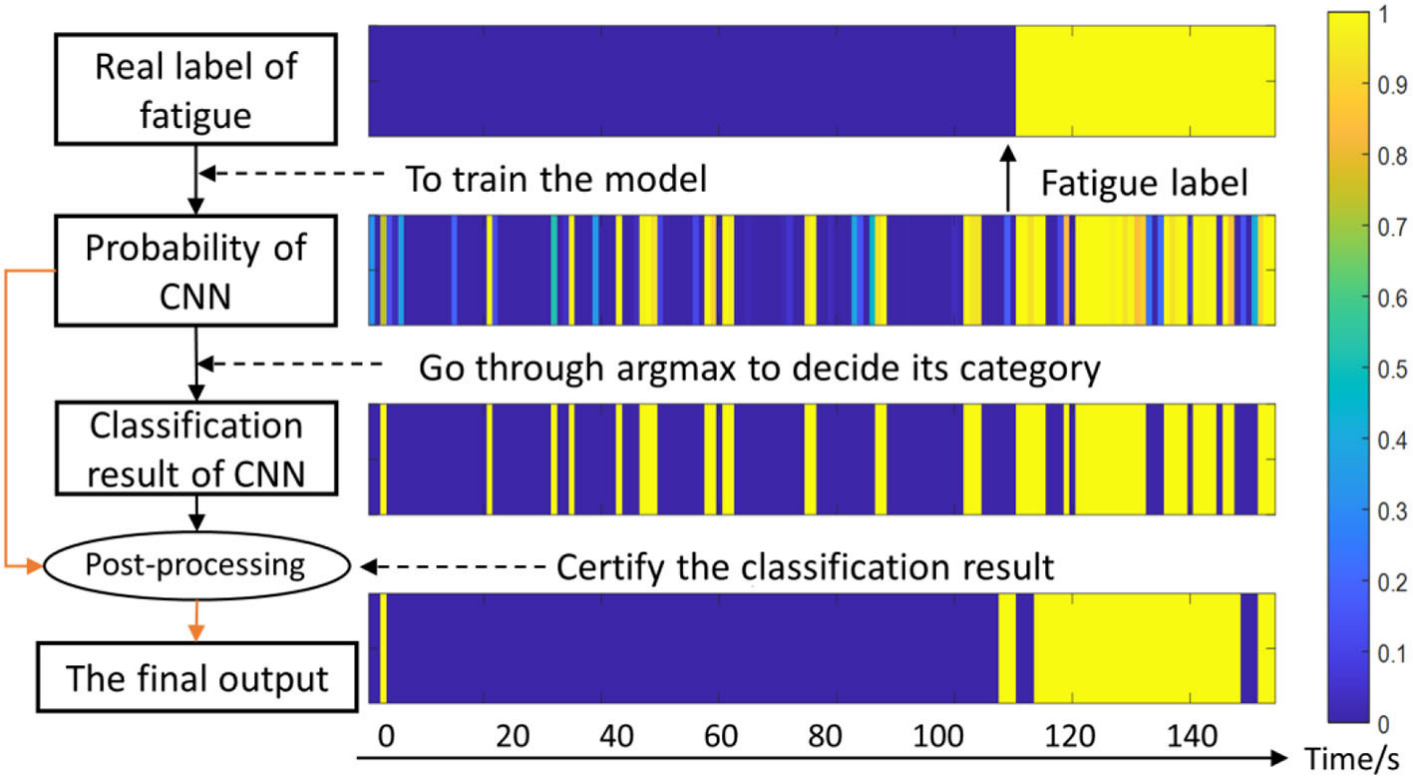
Diagram of the fatigue detection algorithm. The color-bar denotes the possibility of fatigue, for instance, 1 represents totally fatigue and 0 represents non-fatigue. The horizontal axis represents the time series.

Considering one subject's data as example, the effects of post-processing are shown in Figure 7. The preliminary prediction results are the original output of the CNN (the third row), and the probability is the output of the softmax layer (the second row). Then these two results are incorporated in the post-processing layer. Through this approach, the sequential relationship of sEMG is leveraged, and therefore the final accuracy is improved.

### 4.3 Experimental results of different feature extraction schemes

The results of Experiment II are shown in Table 2. First, After Boruta algorithm, the size of features decreases considerably, while the highest accuracies remain stable. Specifically, the decreasing rates of feature size are 64.7, 74.0, and 27% for B, C and D schemes. And the highest accuracies of B, C and D group are 88.61 ± 1.89%, 95.82 ± 1.15% and 95.27 ± 1.14%, respectively, which are very close to the highest accuracies without Boruta algorithm.

**Table 2:** The accuracies of muscle fatigue detection in different feature schemes.

**Method**	**Original features**				**Features after Boruta**			
	**num**	**K** = **3(%)**	**K** = **4 (%)**	**K** = **5 (%)**	**Num**	**K** = **3 (%)**	**K** = **4 (%)**	**K** = **5 (%)**
B	312	86.8 ± 1.01	87.59 ± 1.03	**88.42 ± 1.79**	110	86.53 ± 1.47	**88.61 ± 1.89**	88.14 ± 2.42
C	192	93.23 ± 1.61	94.47 ± 1.51	**94.59 ± 0.99**	50	94.64 ± 1.25	**95.82 ± 1.15**	95.04 ± 0.92
D	48	94.96 ± 1.63	95.16 ± 1.17	**95.54 ± 1.00**	35	94.49 ± 1.94	94.37 ± 1.54	**95.27 ± 1.14**

The bold values indicate the highest accuracy in the “K = 3, K = 4, K = 5” columns of each method.

**Figure 8:**
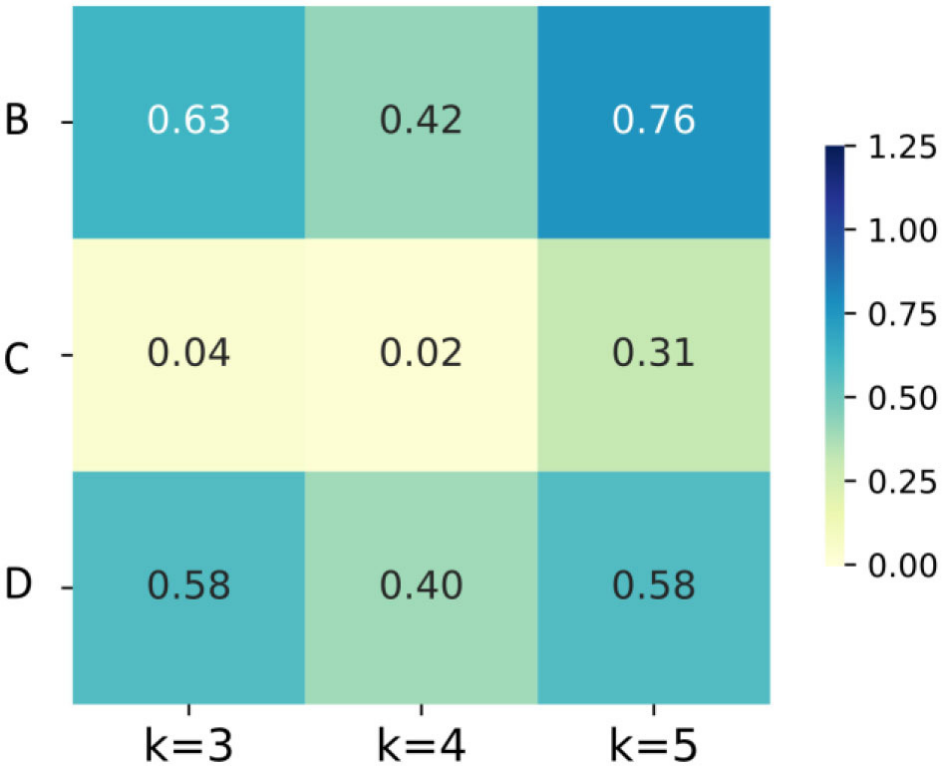
The heatmaps of *P*-values of the accuracies for groups with different K values and different feature schemes.

Second, considering the accuracies of the same dataset (B, C or D) at the same K value before or after Boruta as a couple, *T*-test was conducted to shown the significance of the difference. As illustrated in Figure 8, the differences of accuracies are not significant in B and D, but in the K = 3 and K = 4 group of scheme C, the improvements of accuracy are significant. Third, K = 3 group of scheme C has the least computation expense, applicable time expense and acceptable accuracy. To sum up, the post-processing layer employs the temporal association of sEMG to improve the muscle fatigue detection accuracy, and Boruta algorithm selects the vital features to decrease the computation expense and stabilize the effects of post-processing.

### 4.4. EEG classification results

**Figure 9:**
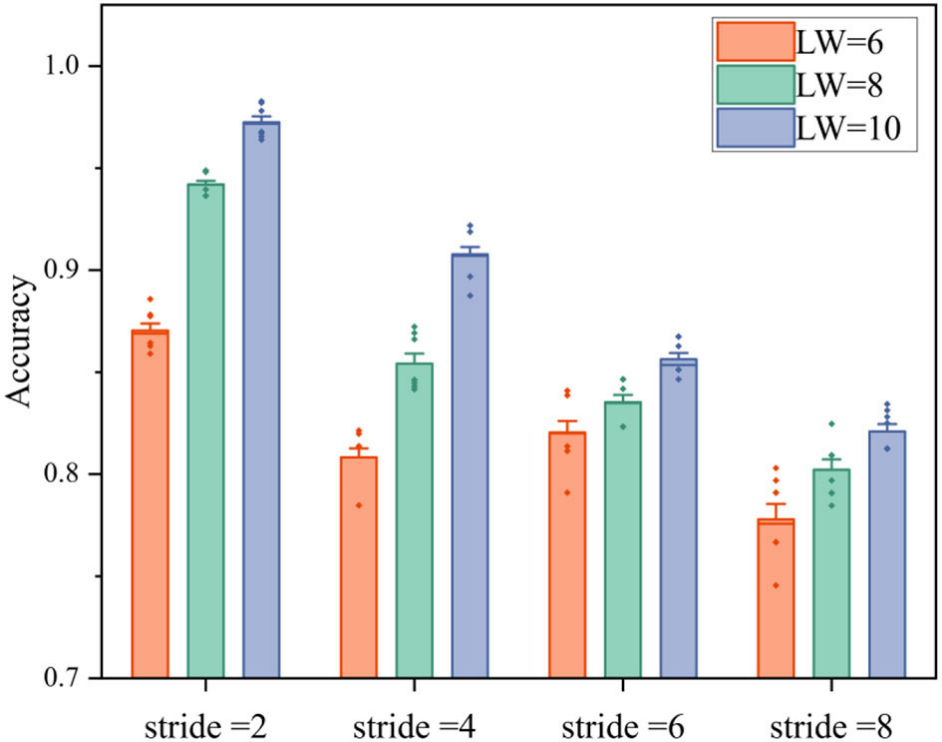
The classification accuracies of movement or non-movement with different strides and window lengths in four fatigue levels. LW is the length of window, and the small rhombuses represent abnormal values.

In Figure 9, the average accuracy of ten times validations of all subjects are illustrated. With the increase of stride, the classification accuracy decreases, meanwhile, the longer length of window generates higher accuracy. Specifically, the lowest accuracy is observed in the group with stride at 8 and LW at 6, which is slightly <80%. When LW = stride = 8, there is no overlapped data in the sliding window, and the accuracy of that group is 80.12 ± 1.1%. Moreover, the highest accuracy achieves up to 97 ± 0.6% in the group where LW = 10 and stride = 2.

According to the results, a longer window and higher overlapping can generate higher accuracy, but will lead to longer time-latency. To balance the requirement of real-time and accuracy, the window length and step length of EEG mode are set as 8 and 2, respectively.

## 5. Discussion

This study proposes a bilateral-controlled wrist rehabilitation system with a switching strategy, aiming to encourage patients to maintain spontaneous rehabilitation and promote the recovery of neurons for individuals with hemiparesis. Notably, previous works on fusion control of sEMG and EEG relied on numerous channels to extract signals, as shown in Table 3, and the devices utilized in these studies were costly and cumbersome, requiring more professional guidance and extended setup time. For instance, Li et al. (2017) used REFA 128 amplifier (TMS International, the Netherlands) with 64-channel EEG+32-channel sEMG; Chowdhury et al. (2019) used g.USBamp amplifier (g.tec, Graz, Austria) in the paper. These amplifiers (cost more than $ 15,000) must be connected to EEG cap with many wires, in this case, there must be assistants to help the patients wear the EEG cap and inject conductive gel into the caps, while the sEMG electrodes must be pasted on the correct muscle as well. In contrast, the rehabilitation system presented in this paper is portable and affordable, consisting of a lightweight exoskeleton and two low-cost, portable signal-acquisition devices, and can be directly used for acquiring signals. Nonetheless, this paper primarily focuses on the rehabilitation system's strategy, with the integration and performance testing to be conducted in subsequent research. Moreover, specific limitations of this system are discussed in the following sections.

**Table 3:** Characteristics in systems that combine EEG and sEMG.

**Research**	**Feature extraction**	**EEG EMG combination**	**Acquisition channel**	**Classification method**	**Category number**	**Classification accuracy**	**Real-time performance**
Li et al. (2017)	Time domain features	Simple fusion	64-ch EEG + 32-ch sEMG	LDA	2-class recognition	80.7–94.2% on single subject	Offline
Chowdhury et al. (2019)	CBPT and CMC	Correlation fusion	3-ch EEG + 2-ch sEMG	SVM	5-class recognition	84.5–92.8% on single subject	Offline
Leeb et al. (2011)	PSD for EEG time features for sEMG	Bayesian fusion	16-ch EEG + 4-ch sEMG	Gaussian classifier	2-class recognition	60.4–92.0% on single subject	Offline
Li et al. (2020)	CSP for EEG time features for sEMG	Hierarchical control	32-ch EEG + 6-ch sEMG	LDA	4-class recognition	73.3–85.3% on single subject	Online
Du et al. (2012)	Wavelet	Sequential control	EEG + EMG + optical fiber	BP network	2-class recognition	62.5–82.5% for EEG 78–89% for sEMG	Online
This paper	PSD for EEG temporal and frequency features for sEMG	Switch control	3-ch EEG 8-ch sEMG	CNN	6-class for sEMG 2-classFor EEG	80.12–97% for EEG 94.49% for sEMG cross subjects	Online

### 5.1. EEG control using a low-cost and portable device

In this study, we present a novel approach using a low-cost EEG band to distinguish spontaneous intention of subjects as manifested by forearm movements and concentration state. This alternative solution is advantageous for rehabilitation, as it enables patients to fulfill consecutive training and maintain voluntary participation (Kwakkel et al., 2004; Wang et al., 2013). While most previous studies have focused on extracting EEG signals from corresponding regions of the motor nerves (Lalitharatne et al., 2013; Chowdhury et al., 2019; Li et al., 2019), this research employed forehead EEG signal to reflect the user's motor intention, and this method has been studied in a recent paper (Liu et al., 2022).

The paradigm presented in this paper uses EEG activation from both movement and visual attention, while reducing the classification into two categories (move or still), resulting in higher accuracy for EEG control mode. Another reason for designing this control paradigm is to prepare for incorporating MYO information and EEG signals in the future. The position information can be detected through the MYO Armband's Inertial Measurement Unit (IMU), and is not affected by muscle fatigue. We chose not to use computer vision (CV) to detect motions because it is prone to be affected by lighting, camera quality, and obstructions. In contrast, EEG directly reflects brain activity and is not affected by these factors. Furthermore, the wireless EEG headband used in this paper is not limited by the range of the camera, which provides greater portability. However, the application of this strategy is for the hemiparesis of the early stage when their damaged wrist is paretic and not able to move, in the future, a resistance training mode through incorporating force sensor into the exoskeleton would be studied for the latter stage of recovery.

### 5.2. SEMG fatigue detection and its limitations

In this study, a novel approach for detecting muscle fatigue using a low-cost EMG armband is proposed. By introducing a non-linear bias through a simple equation (1), the classification accuracy is significantly improved, as demonstrated in the results. Furthermore, the Boruta algorithm (Kursa and Rudnicki, 2010) is utilized to reduce the feature size and improve stability of the post-processing. Instead of selecting the feature set for a specific model, the Boruta algorithm can filter out all the feature sets that are correlated with the dependent variable, thus the stabilization and generalization are better. Note that although all feature samples were employed in the experiment to select the optimal features, in practical application, a smaller segment of signals can be used in finding the index of the optimal features prior to the formal detection, so that the processing of sEMG in real-time detection does not include Boruta algorithm. For each detection, the fatigue detection program acquires 0.75 s sEMG as the data basis, and the feature extraction needs < 0.05 s, while the CNN and post-processing procedures can be completed within 0.1 s. Therefore, the fatigue detection program can output a result at 1 Hz. Although this model is run on a laptop with R7 5800 CPU and RTX 3050 GPU, the portability is not affected because the command is sent by Bluetooth which is effective to cover the daily using in regular house.

There is still room for improvement in this approach. Firstly, the current method can only accomplish two-class classification, and a fatigue index should be developed to define the fatigue level. The post-processing method only considers the original predicted results to rectify the output, and dynamic rectification involving the already corrected neighboring results should be investigated and tested in future work. Secondly, only four schemes for feature extraction are used in this study, which may not be the optimal options. Hence, more features need to be involved and tested in the future. Third, In the future, the CNN model could be replaced by machine learning methods like SVM in order to support the embedded system. Finally, the validation of detection model is based on healthy young subjects of which the sEMG signals is different from those of the old subjects. In the future, the sEMG signal of old subjects should be acquired to train a new model, in which the parameters of Boruta algorithm and CNN network are different.

## 6. Conclusion

In this study, a novel, low-cost and portable rehabilitation system for wrist recovery is presented. The system incorporates a sEMG-EEG combined strategy to promote prolonged consecutive training, with a key contribution being the muscle fatigue detection method. The use of the Boruta algorithm and post-processing method improves fatigue detection accuracies for four motions. Additionally, an alternative control method using EEG and a CNN model achieves high accuracy in detecting motion intention.

However, this paper mainly focuses on the control strategy, and future work should include integration and validation of the system. Moreover, there is room for improvement, such as incorporating IMU with EEG mode, and developing a method for recognizing and defining graded muscle fatigue. Overall, this study offers an effective control strategy for rehabilitation robots that has the potential to improve patient outcomes.

## Data availability statement

The datasets presented in this study can be found in online repositories. The names of the repository/repositories and accession number(s) can be found below: https://ieee-dataport.org/documents/fatigue-semg-data.

## Ethics statement

The studies involving human participants were reviewed and approved by Institutional Review Board of Xi'an Jiaotong University. The patients/participants provided their written informed consent to participate in this study. Written informed consent was obtained from the individual(s) for the publication of any identifiable images or data included in this article.

## Author contributions

Conceptualization and methodology: SY and ML. Supervision and funding acquisition: ML. Experiment design and data analysis: SY, ML, and JW. Investigation: SY, JW, ZS, and BH. Writing: SY, ML, JW, JX, and GX. All authors contributed to the article and approved the submitted version.
